# Supplementary material for: Biosorption of Pb(II) Using Natural and Treated Ardisia compressa K. Leaves: Simulation Framework Extended through the Application of Artificial Neural Network and Genetic Algorithm
Source: Molecules. 2023 Aug 31;28(17):6387. doi: 10.3390/molecules28176387 (PMC10490334; doi:10.3390/molecules28176387)
Supplement: Supplementary file 1 [file molecules-28-06387-s001.zip › molecules-2527849-supplementary.pdf]

Supplementary Material

Error functions analysis

In addition to the regression coefficient (R^2), the nonlinear forms of kinetic and isotherms models were evaluated against the experimental data using the error functions to test the adequate model for the adsorption system. For this purpose, the average relative error (ARE), the sum of square error (SSE), normalized standard deviation, Δq (%), Chi-square test (χ^2), the sum of absolute error (EABS), and root mean square error (RMSE) were calculated using the equations 9, 10, 11, 12, 13, and 14, respectively. These error functions were the most used in the literature [17].

$$ARE = \sum_{i=1}^{n} \frac{1}{N} \frac{\left|(q_{e,exp} - q_{e,cal}\right|}{q_{e,exp}} * 100 \qquad (Eq. 9)$$

$$SSE = \sum_{i=1}^{n} (q_{e,exp} - q_{e,cal})^2 \qquad (Eq. 10)$$

$$\Delta q(\%) = 100 \sqrt{\frac{1}{N-1} \sum_{i=1}^{n} (\frac{q_{e,exp} - q_{e,cal}}{q_{e,exp}})^2} \qquad (Eq. 11)$$

$$\chi^2 = \sum_{i=1}^{n} \frac{(q_{e,exp} - q_{e,cal})^2}{q_{e,cal}} \qquad (Eq. 12)$$

$$EABS = \sum_{i=1}^{n} \left|(q_{e,exp} - q_{e,cal}\right| \qquad (Eq. 13)$$

$$RMSE = 100 \sqrt{\frac{1}{N-2} \sum_{i=1}^{n} (q_{e,exp} - q_{e,cal})^2} \qquad (Eq. 14)$$

where $q_{e,exp}$ (mg/g) is the experimental adsorption capacity obtained from the Eq. (1) and $q_{e,cal}$ (mg/g) is the calculated adsorption capacity obtained from the nonlinear kinetic models (Pseudo-first order and pseudo-second order) or isotherm models (Langmuir and Freundlich), n is the number of experimental data points.
